# Transcriptome and miRNAs Profiles Reveal Regulatory Network and Key Regulators of Secondary Xylem Formation in “84K” Poplar

**DOI:** 10.3390/ijms242216438

**Published:** 2023-11-17

**Authors:** Huilin Wang, Pan Zhao, Yumei He, Yuting Su, Xinyi Zhou, Huihong Guo

**Affiliations:** State Key Laboratory of Tree Genetics and Breeding, College of Biological Sciences and Biotechnology, Beijing Forestry University, No. 35, Tsing Hua East Road, Haidian District, Beijing 100083, China; huilinwang21@163.com (H.W.); zhaopan11250528@163.com (P.Z.); yumeihe2021@163.com (Y.H.); suyt6920@163.com (Y.S.); xyzhoubot@163.com (X.Z.)

**Keywords:** “84K” poplar, secondary xylem formation, transcriptome, miRNAs, transcriptional regulation

## Abstract

Secondary xylem produced by stem secondary growth is the main source of tree biomass and possesses great economic and ecological value in papermaking, construction, biofuels, and the global carbon cycle. The secondary xylem formation is a complex developmental process, and the underlying regulatory networks and potential mechanisms are still under exploration. In this study, using hybrid poplar (*Populus alba* × *Populus glandulosa* clone 84K) as a model system, we first ascertained three representative stages of stem secondary growth and then investigated the regulatory network of secondary xylem formation by joint analysis of transcriptome and miRNAs. Notably, 7507 differentially expressed genes (DEGs) and 55 differentially expressed miRNAs (DEMs) were identified from stage 1 without initiating secondary growth to stage 2 with just initiating secondary growth, which was much more than those identified from stage 2 to stage 3 with obvious secondary growth. DEGs encoding transcription factors and lignin biosynthetic enzymes and those associated with plant hormones were found to participate in the secondary xylem formation. MiRNA-target analysis revealed that a total of 85 DEMs were predicted to have 2948 putative targets. Among them, PagmiR396d-*PagGRFs*, PagmiR395c-*PagGA2ox1*/*PagLHW*/*PagSULTR2*/*PagPolyubiquitin* 1, PagmiR482d-*PagLAC4*, PagmiR167e-*PagbHLH62*, and PagmiR167f/g/h-*PagbHLH110* modules were involved in the regulating cambial activity and its differentiation into secondary xylem, cell expansion, secondary cell wall deposition, and programmed cell death. Our results give new insights into the regulatory network and mechanism of secondary xylem formation.

## 1. Introduction

In trees, stem growth and development go through two phases, i.e., primary growth and secondary growth. The shoot apical meristem drives primary growth, i.e., longitudinal elongation, and produces primary vasculature. The onset of secondary growth, i.e., radial thickening, is accompanied by reactivation of the fascicular cambium within primary vascular bundles and dedifferentiation of parenchymal cells located in the interfascicular region (interfascicular cambium); a complete ring of cambium, which is connected by the fascicular and interfascicular cambiums, continuously divides and differentiates into secondary xylem inwards and secondary phloem outwards to expand girth [1]. The secondary xylem is mainly composed of vessels and fibers, which bear water and nutrition transport as well as support functions necessary for tree growth [2]. Notably, the secondary xylem (also called wood) in the stem, as the main source of tree biomass, is of great economic and ecological value in papermaking, construction, biofuels, and the global carbon cycle [3,4]. The formation of secondary xylem is a complex developmental process, including cambial differentiation into secondary xylem, cell expansion, secondary cell wall deposition, and programmed cell death (PCD) [3]. However, the molecular network underlying the secondary xylem formation is still being explored.

Transcriptome sequencing can quickly obtain transcripts of different tissues or organs of plants at different developmental stages and identify key genes related to plant growth and development, which is very helpful for analyzing the regulatory networks and potential mechanisms underlying plant growth and development [5]. In earlier studies, we obtained some information about the potential mechanisms of plant secondary growth through a transcriptomic study of model plant *Arabidopsis* [6,7]. However, the limited extent of secondary growth of *Arabidopsis* largely limits our understanding of plant secondary growth [8]. In recent years, transcriptomic analysis in the perennial woody tree species, such as poplar, has enhanced our understanding of the regulatory networks and potential mechanisms underlying secondary growth and secondary xylem development [8]. For example, differentially expressed genes (DEGs) associated with stem primary and secondary growth were identified through the RNA-seq analysis of *Populus deltoides* × *Populus euramericana cv* ‘*Nanlin895*’ [9]; a large number of DEGs associated with secondary cell wall formation and maintenance of cambial activity were identified in stems with significant secondary growth in *P. alba* × *P. glandulosa* clone BH using transcriptomic sequencing [10]. In view of the fact that secondary xylem is the main part of secondary structure derived from secondary growth, several studies have focused on changes in the transcriptional profile during secondary xylem development in poplar [11,12]. For example, it was found that most DEGs were associated with secondary cell wall thickening and PCD through pairwise transcriptomic analysis of the secondary xylem from five black poplar cultivars [11]. Similarly, a transcriptional regulatory network for cell wall biosynthesis and PCD was constructed by analyzing the transcriptomic profiles of secondary xylem at different developmental stages in *P. tomentosa* [12]. Although some advances have been made in the studies of secondary growth and secondary xylem development in previous studies, the transcriptional profile of the key transition period from primary growth to secondary growth has not been accurately analyzed due to the lack of accurate ascertainment of the transition period in poplar stems. Moreover, as mentioned earlier, because secondary xylem formation involves a series of complex developmental events, the regulatory networks and potential mechanisms underlying the events of cambial activity and its differentiation into secondary xylem, cell expansion, and PCD still need to be explored.

MicroRNAs (miRNAs) are a class of endogenous non-coding small RNAs composed of 18–24 nucleotides, which are involved in regulating multiple processes of plant growth and development at the post-transcriptional level by binding to the reverse complementary sequences of target mRNAs, degrading the target mRNAs or inhibiting the translation of target mRNAs [2,13]. Small RNA sequencing (RNA-seq) has become a useful tool for the rapid discovery of miRNAs and can be used to identify important miRNAs associated with plant growth and development [14]. For example, a total of 85 xylem-specific differentially expressed miRNAs (DEMs) were identified by small RNA-seq analyses of xylem, phloem, and leaves in hybrid poplar (*P. alba* × *P. glandulosa*), which were thought to be potentially involved in regulating secondary xylem formation [15]; 75 DEMs associated with stem secondary growth were identified by small RNA-seq in *P. deltoids* [16]. However, little is currently known about which miRNAs are involved in regulating the transition from primary to secondary growth in poplar stems and which genes these miRNAs target are involved in these developmental processes.

To address the above-mentioned issue, we investigated the regulatory network and potential mechanism underlying the onset of secondary xylem formation and its subsequent development in hybrid poplar (*P. alba* × *P. glandulosa* clone 84K) by joint analysis of transcriptome and miRNA. Our findings will provide new insights into the molecular mechanism of secondary xylem formation in trees.

## 2. Results

### 2.1. Anatomical Observations of Three Representative Developmental Stages of “84K” Poplar Stems

**Figure 1:**
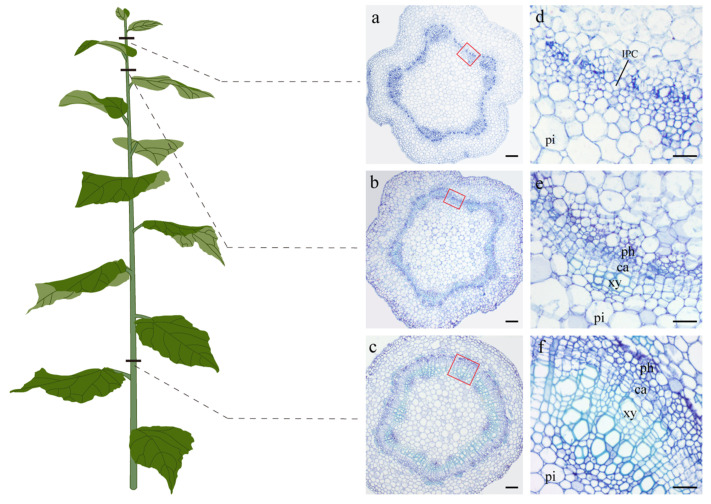
Cross-sections of three representative developmental stages of “84K” poplar stems. (**a**,**d**) Secondary growth had not yet been initiated in the 2nd internode. (**b**,**e**) Secondary growth had just been initiated in the 3rd internode. (**c**,**f**) Obvious secondary growth was observed in the 10th internode. (**d**–**f**) are enlarged views of the red squares in (**a**–**c**), respectively. IPC: interfascicular parenchymal cells, ph: phloem, ca: cambium, xy: xylem, pi: pith. Scale bars, 100 μm.

Through observing the anatomical structures of the 1st–10th internodes of “84K” poplar stems, it was found that the secondary growth had not yet been initiated in the 1st–2nd internodes and had just been initiated in the 3rd–4th internodes; in the subsequent 5th-10th internodes, secondary growth was continuously enhanced, and the vascular tissue produced by cambium gradually increased. Therefore, we defined the developmental periods without initiating secondary growth, with just initiating secondary growth, and with obvious secondary growth as stage 1, stage 2, and stage 3, respectively. In stage 1, vascular bundles with fascicular cambium were arranged in a discontinuous ring along the stem circumference, and interfascicular cambium had not yet been formed (Figure 1a,d); in stage 2, the fascicular cambium was connected with the interfascicular cambium to form a complete annular cambium, which produced a small amount of secondary vascular tissues by its division and differentiation inwards and onwards (Figure 1b,e); in stage 3, more secondary vascular tissues, in particular, secondary xylem cells were produced by the cambium (Figure 1c,f).

### 2.2. Transcriptome Analysis during Secondary Growth of “84K” Poplar Stems

#### 2.2.1. RNA Sequencing

Through transcriptome sequencing of nine RNA libraries from the above-mentioned three developmental stages of “84K” poplar stems, a total of 581,038,394 cleaned reads were obtained after removing the reads containing adapter and poly-N as well as low-quality reads. The GC contents ranged from 44.24% to 44.63%, and Q30’s were above 92.96%, respectively (Table S1). The cleaned reads were mapped to the *P. trichocarpa* genome (reference genome), and the mapped reads accounted for 82.96% of the total cleaned reads (Table S1). A principal component analysis (PCA) revealed that the samples from three stages were significantly separated, and the three replicates from the same stage were clustered together (Figure S1). These results indicated that sample collection and reference genome selection are appropriate, and sequencing quality is qualified for subsequent analysis.

#### 2.2.2. Identification and Functional Annotation of Differentially Expression Genes

**Figure 2:**
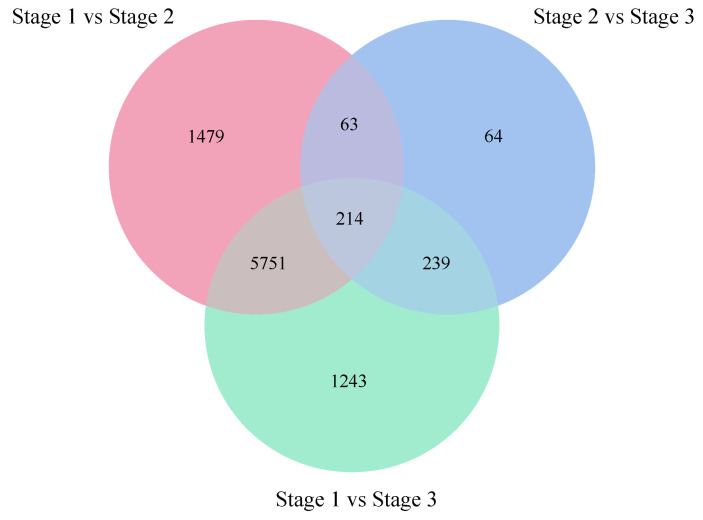
Numbers of differentially expressed genes (DEGs) in stage 1 versus stage 2, stage 2 versus stage 3, and stage 1 versus stage 3.

To identify DEGs that were associated with the secondary growth of stems, we performed pairwise comparisons among the three developmental stages of “84K” poplar stems. In total, 9055 DEGs were identified based on the criteria of FDR < 0.01 and |log2 fold change| ≥ 1 (Figure 2; Table S2). The Venn diagram showed that 214 DEGs were present in all three stages, while 1479 DEGs, 64 DEGs, and 1243 DEGs were specifically expressed in stage 1 versus stage 2, stage 2 versus stage 3, and stage 1 versus stage 3, respectively (Figure 2).

Through the functional annotation of the above-mentioned DEGs, it was found that the DEGs in stages 1 versus stage 2 were mainly enriched in phenylpropanoid biosynthesis, plant hormone signal transduction, starch and sucrose metabolism, carbon metabolism, amino acid biosynthesis, and plant-pathogen interaction. The DEGs enrichment pathways in stages 2 versus stages 3 and stages 1 versus stages 3 were similar to those in stages 1 versus stage 2 (Figure S2).

#### 2.2.3. DEGs Related to the Secondary Xylem Formation

**Figure 3:**
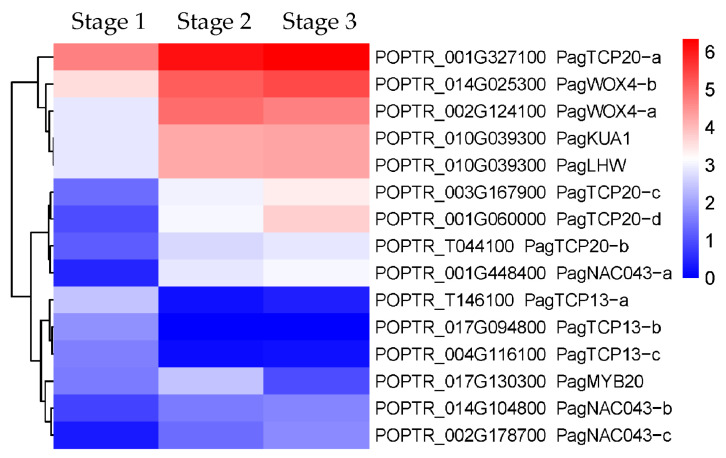
Heatmap of differentially expressed genes (DEGs) involved in secondary xylem formation in “84K” poplar stems. *PagNAC043*, the homolog of *AtNST1*/*2* and *AtSND1*; *PagMYB20*, the homolog of *AtMYB46*/*83*.

DEGs encoding members of the WOX, TCP, NAC, MYB, and bHLH transcription factor (TF) families were identified during the secondary growth of “84K” poplar stems. From stage 1 to stage 2, 58 DEGs encoding MYB TFs (40 up-regulated/18 down-regulated), 37 DEGs encoding NAC TFs (31 up-regulated/6 down-regulated), 34 DEGs encoding bHLH TFs (24 up-regulated/10 down-regulated), 15 DEGs encoding TCP TFs (6 up-regulated/9 down-regulated), and two DEGs encoding WOX TFs (up-regulated) were identified; from stage 2 to stage 3, 10 DEGs encoding bHLH TFs (5 up-regulated/5 down-regulated), 9 DEGs encoding MYB TFs (up-regulated), and 3 DEGs encoding NAC TFs (2 up-regulated/1 down-regulated) were identified (Table S3). These DEGs were mainly enriched in the developmental transition from stage 1 to stage 2. In trees, the main structure produced in the secondary growth of stems is the secondary xylem, WOX4 of the WOX family, TCP13/20 of the TCP family, LHW of the bHLH family, MYB1R1-like (KUA1) of the MYB family, NST1/2 and SND1 of the NAC family, and MYB46/83 of the MYB family have been confirmed to be involved in regulating secondary xylem formation [2,17,18,19]. The expression of homologs encoding WOX4, TCP20, LHW, KUA1, NST1/2/SND1, and MYB46/83 was significantly up-regulated from stage 1 to stage 2 of “84K” poplar stems, while the expression of homologs encoding TCP13 was significantly down-regulated; however, there was no significant difference in their expression levels from stage 2 to stage 3 (Figure 3).

**Figure 4:**
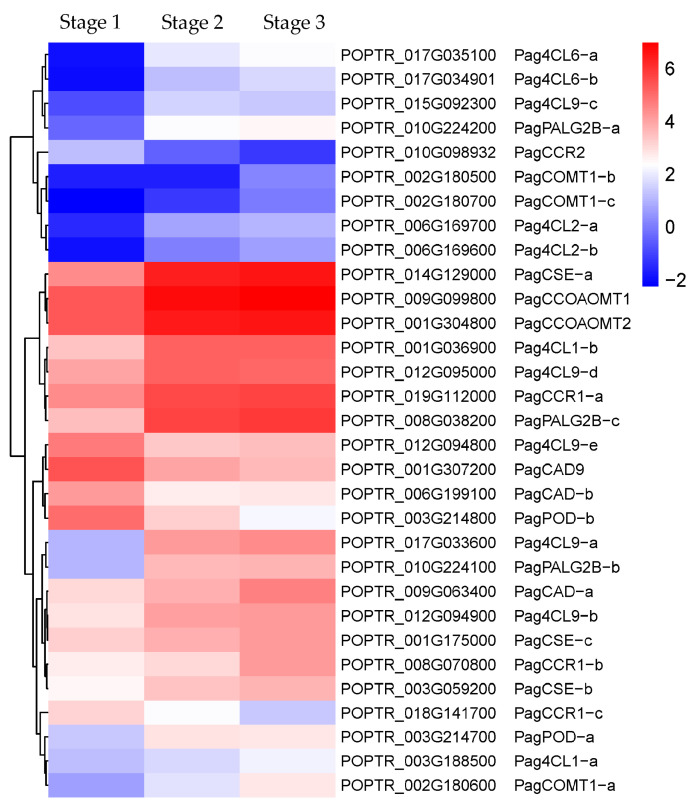
Heatmap of differentially expressed genes (DEGs) involved in phenylpropanoid biosynthesis in “84K” poplar stems.

The lignins synthesized by the phenylpropane biosynthetic pathway are the main component of the cell walls of the secondary xylem. Therefore, the phenylpropane pathway is closely related to the secondary growth of tree stems, especially to the formation of the secondary xylem [20]. In this study, 25 DEGs from stage 1 to stage 2 were assigned to the phenylpropane pathway (20 up-regulated/5 down-regulated), and only two up-regulated DEGs from stage 2 to stage 3 were assigned to the phenylpropane pathway (Table S4). These DEGs primarily encode enzymes including phenylalanine ammonia-lyase (PAL), 4-coumarate CoA: ligase (4CL), cinnamoyl-CoA reductase (CCR), cinnamyl alcohol dehydrogenase (CAD), peroxidase (POD), caffeoyl-coenzyme A O-methyltransferase (CCoAOMT), caffeic acid 3-O-methyltransferase (COMT), and caffeoyl shikimate esterase (CSE) (Figure 4).

**Figure 5:**
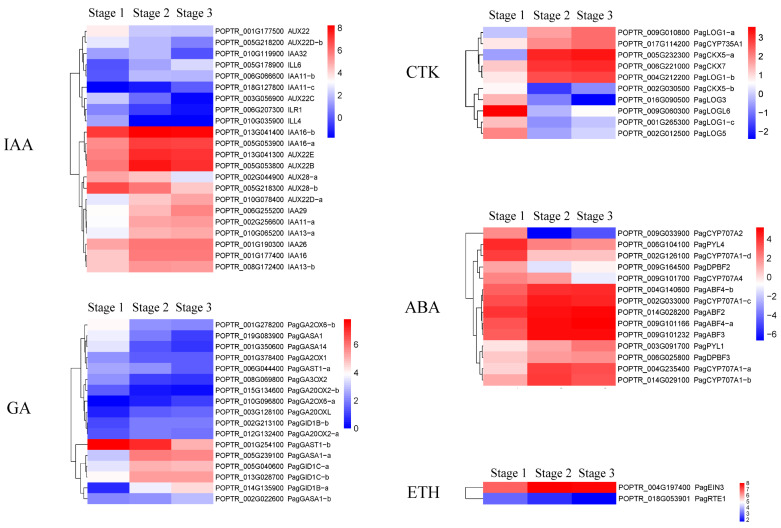
Heatmap of plant hormones-related differentially expressed genes (DEGs) involved in secondary xylem formation of “84K” poplar stems.

Plant hormones have been proven to be involved in triggering xylem differentiation and stem growth in trees [21]. In this study, a large number of DEGs associated with the biosynthesis and signal transduction of plant hormones, including auxin (IAA), gibberellin acid (GA), cytokinin (CTK), ethylene (ETH), abscisic acid (ABA), and brassinosteroid (BR) were identified (Figure 5). The largest number of DEGs were enriched in the IAA signaling pathway, with 13 DEGs significantly up-regulated and four significantly down-regulated in stages 1 to 2, and only three significantly down-regulated DEGs identified in stages 2 to 3 (Figure 5). The vast majority of DEGs associated with GA, CTK, ETH, ABA, and BR biosynthesis, metabolism, and signal transduction were significantly up-regulated in stages 1 to 2, but there was no significant difference in stages 2 to 3 (Figure 5).

### 2.3. Analysis of miRNAs and Their Targets during Secondary Growth of “84K” Poplar Stems

#### 2.3.1. Sequencing of Small RNAs and Identification of miRNAs

A total of 51,232,372 cleaned reads were obtained by sequencing nine small RNA libraries from the three developmental stages of “84K” poplar stems, and 32,446,284 cleaned reads were mapped to the reference genome (Table S5). Through comparing mapped reads with miRNAs in the miRBase (v22) database, 293 known miRNAs were identified; in the remaining unmapped reads, 136 novel miRNAs were identified using miRDeep2 software (v2.0.5) (Table S6).

#### 2.3.2. DEMs and Their Targets Related to Secondary Xylem Formation

**Figure 6:**
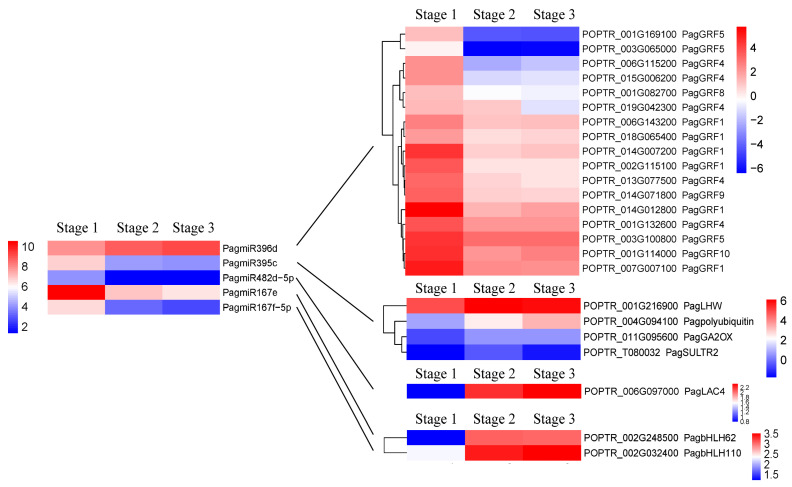
Heatmap of differentially expressed miRNAs (DEMs) with their targets involved in secondary xylem formation of “84K” poplar stems.

MiR396d and miR395c have been shown to be involved in regulating the secondary xylem development as positive and negative regulators in poplar [22,23]. In this study, we observed that in stages 1 to 2, PagmiR396d was significantly up-regulated, whereas PagmiR395c was significantly down-regulated; however, there was no significant difference in their expression levels in stages 2 to 3 (Figure 6). For PagmiR396d, a total of 123 putative target genes were identified (Table S7), of which *PagGRF1*/*4*/*5*/*8*/*9*/*10*, *PagCSP41A*, *PagPFC1*, *PagBAC1*, *PagRD21A*, *PagEMB975*, *PagCHLD* and *At4g02110* showed significantly opposite expression trends with PagmiR396d (Figure 6). For PagmiR395c, 25 putative target genes were identified (Table S7), in which *PagLHW*, *PagSULTR2*, *PagPolyubiquitin 1*, *PagGA2OX1*, and *PagRKS1* showed significantly opposite expression trends with PagmiR395c (Figure 6). In addition, PagmiR428d and its putative target gene *PagLAC4*, and PagmiR167e/f/g/h and their putative target genes *PagbHLH62*/*110* were found to show significantly opposite expression trends in stages 1 to 2 (Figure 6).

### 2.4. RT-qPCR Validation of DEMs and Their Targets

To validate the reliability of small RNAs and mRNAs sequencing, five DEMs and their target genes were selected for RT-qPCR validation. The RT-qPCR results were consistent with the sequencing data (Figure S3), supporting the reliability of sequencing data.

## 3. Discussion

### 3.1. Transcription Factors Regulate Secondary Xylem Formation

#### 3.1.1. Regulation of Cambial Activity and Its Differentiation into Xylem

**Figure 7:**
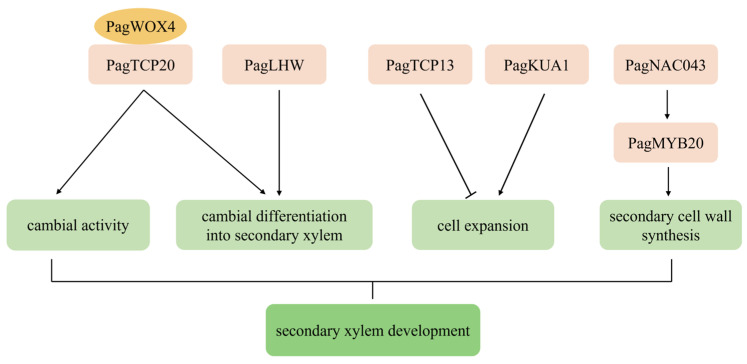
A model for the roles of transcription factors in regulating the secondary xylem formation of “84K” poplar stems. Transcription factors are shown in pink and yellow boxes.

WOX4, TCP20, and LHW transcription factors can regulate the division and differentiation of vascular stem cells [19,24,25]. RNAi-mediated silencing of *PtrWOX4a*/*b* gene reduced cambial cell division activity, resulting in a significant decrease in secondary xylem but did not significantly affect the secondary phloem development in *P. trichocarpa* stems [25], suggesting that WOX4 is a key factor in regulating cambial activity and its differentiation into secondary xylem. Further studies revealed that the PtrWOX4 controls cambial activity by interacting with PtrTCP20, which promotes the differentiation of cambium into secondary xylem by activating downstream transcription factors [19]. In this study, PagWOX4 and PagTCP20 were significantly up-regulated in the secondary growth of “84K” poplar stems (Figure 3), indicating that the functions of *WOX4* and *TCP20* homologs are conserved in *Populus*. In addition, the mutation of *AtLHW*, a member of the *bHLH* transcription factor gene family, led to the halving of the number of protoxylem cells in the primary roots of *Arabidopsis*, and thus LHW is believed to control early xylem development in the roots by regulating the size of the vascular stem cell pool [24]. In this study, *PagLHW* was found to be significantly up-regulated in the secondary growth of “84K” poplar stems (Figure 3), indicating that there was a certain functional differentiation of *LHW* from *Arabidopsis* to poplar, and *PagLHW* may be involved in regulating the cambial differentiation into secondary xylem in “84K” poplar stems (Figure 7).

#### 3.1.2. Regulation of Xylem Cell Expansion and Secondary Cell Wall Formation

KUA1 of the MYB family and TCP13 of the TCP family were found to be involved in regulating cell expansion of xylem cells. In *Arabidopsis*, AtKUA1 positively regulated the cell expansion [18]; however, AtTCP13 negatively regulated the cell expansion [26]. This study found that *PagKUA1* was significantly up-regulated whereas *PagTCP13* was significantly down-regulated during the secondary growth of “84K” poplar stems (Figure 3), which suggests that *PagKUA1* and *PagTCP13* likely regulate the cell expansion of secondary xylem in “84K” poplar stems as positive and negative regulators, respectively (Figure 7).

In the NAC family, NST1/2 and SND1 serve as the first-level master switches to turn on the secondary cell wall biosynthesis network and regulate the secondary cell wall deposition of xylem cells [27,28]. In *Arabidopsis*, AtSND1, AtNST1, and AtNST2 can activate the expression of *AtMYB46* and *AtMYB83*, which are the second-level master switches in the biosynthesis of secondary cell walls, and then AtMYB46 and AtMYB83 further activate the expression of downstream target genes to regulate the development of secondary cell walls of xylem [19,28,29]. In this study, we found that both *PagNAC043* (the homolog of *AtSND1*/*AtNST1*/*2*) and *PagMYB20* (the homolog of *AtMYB46*/*AtMYB83*) were significantly up-regulated in the secondary growth of “84K” poplar stems (Figure 3; Table S8), and thus it is suggested that there is a similar regulatory mechanism in poplar and *Arabidopsis*, that is, the up-regulation of *PagNAC043* expression could activate *PagMYB20* expression, and then the *PagMYB20* further activates the expression of downstream genes to participate in regulating the cell wall formation of secondary xylem in “84K” poplar stems (Figure 7).

#### 3.1.3. Lignin Biosynthesis-Related Enzymes Genes Regulate Secondary Xylem Formation

The secondary xylem is mainly composed of vessels and fibers, and these dead cells possess significantly thickened secondary cell walls rich in lignins [2]. The content and types of lignins deposited in the secondary cell walls have a significant effect on wood properties [30]. Lignins are formed by the polymerization of three units: *p*-hydroxyphenyl (H), guaiacyl (G), and syringyl (S) [20], which are derived from three hydroxycinnamyl alcohol precursors of the phenylpropanoid pathway, namely *p*-coumaryl alcohol, coniferyl alcohol, and sinapyl alcohol [20,31]. PAL, as the first key speed-limiting enzyme in the phenylpropanoid pathway, catalyzes the conversion of phenylalanine to cinnamic acid, which triggers the subsequent secondary metabolism of phenylpropanoid pathway to produce lignin units [32,33]. 4CL, CCR, and CAD are key enzymes that catalyze the formation of the three hydroxycinnamyl alcohol precursors of *p*-coumaryl alcohol, coniferyl alcohol, and sinapyl alcohol, while POD catalyzes the three precursors to form H, G, and S units of lignin [32,34], where G unit can be converted into S unit by CCoAOMT [35]. In *Arabidopsis*, At4CL can form a bypass pathway of monolignol biosynthesis together with AtCSE to participate in the synthesis of lignin H and S units [35,36]. In “84K” poplar, CRISPR-knockout of *PagCSE1* or *PagCSE2* reduced the content of the S lignin unit, resulting in the collapse of the xylem vessels [37], and a similar phenomenon was found in *Arabidopsis* [36]. COMT is also involved in the biosynthesis of S-lignin, and the S-lignin biosynthesis was found to be inhibited in PvCOMT-RNAi transgenic switchgrass plants [38]. In this study, the genes encoding PagPAL, Pag4CL, PagCCR, PagCAD, PagPOD, PagCCoAOMT, PagCOMT, and PagCSE were generally significantly up-regulated in stages 1 to 2 during the secondary growth of the “84K” poplar stems (Figure 4), suggesting that these genes regulate the formation of cell walls of secondary xylem at the initial stage of secondary growth by participating in lignin biosynthesis in poplar.

#### 3.1.4. Plant Hormones Regulate Secondary Xylem Formation

Plant hormones have been demonstrated to be involved in regulating cambial activity and xylem development in plants [39]. The interruption of auxin supply from the shoot apical meristem to the stem downward led to a cessation of radial growth derived from cambial stem cells in *Arabidopsis* [40,41], and a deficiency of exogenous auxin in pine explants led to the differentiation of cambium into parenchymal cells instead of vascular cells [42], indicating that auxin is required for the maintenance of cambial activity and its differentiation into xylem cells. In hybrid poplar (*P. tremula* × *tremuloides*), bioactive GA peaks were detected in the developing xylem [43]. A recent study further confirmed that GA, together with auxin, plays crucial roles in stimulating cambial activity in *P. tomentosa* [44]. CK, ETH, and ABA are also involved in the regulation of cambial activity in poplar. An inhibition of CK signaling reduced the number of cambium cells in hybrid poplar (*P. tremula* × *tremuloides*), whereas an increase in CK biosynthesis increased cambial cell division [43]. Also, in *P. tremula* × *tremuloides*, exogenous ETH applied or endogenous ETH induced by leaning stimulated cambial cell division and secondary xylem formation [45]. The exogenous applications of ABA and BR have also been found to enhance the cambial activity and xylem development in hybrid poplar (*P. nigra* × *P. maximowiczii*) and pine (*Pinus massoniana*), respectively [21,46]. In this study, most of the DEGs associated with IAA, GA, CTK, ETH, ABA and BR biosynthesis, metabolism, and signal transduction were significantly up-regulated in the secondary growth of “84K” poplar stems (Figure 5), which suggests that these DEGs are involved in the secondary xylem formation by positively regulating the cambial activity and its differentiation into secondary xylem in “84K” poplar.

### 3.2. MiRNAs and Their Targets Regulate Secondary Xylem Formation

#### 3.2.1. PagmiR396d-PagGRFs Modules

**Figure 8:**
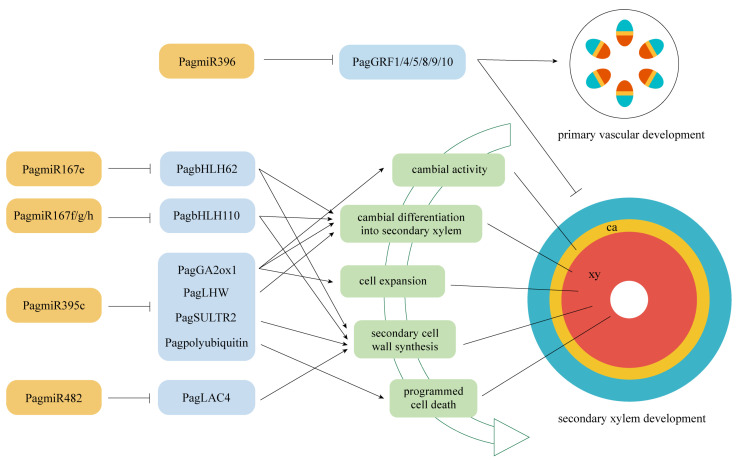
A model for the roles of miRNAs and their targets in the secondary xylem formation of “84K” poplar stems. MiRNAs and their targets are shown in yellow and blue boxes, respectively. “→” represents promotion, while “⊥” stands for suppression. xy: xylem, ca: cambium.

A recent study showed that in *P. tomentosa*, PtoTCP20 activated the PtomiR396d precursor gene, and thereafter, PtomiR396d positively regulated the differentiation of secondary xylem by inhibiting the expression of its target gene *PtoGRF15*, resulting in increased wood production [22]. Given that both *PagMIR396d* and *PagTCP20* were significantly up-regulated in the secondary growth of “84K” poplar stems (Figure 6), and a putative target gene *PagGRF1* (a homolog of *PtoGRF15*) of PagmiR396d was significantly down-regulated (Figure 6), suggesting that the *PagTCP20*-PagmiR396d-*PagGRF1* module regulates the secondary xylem formation in “84K” poplar stems, and the regulatory module is conserved in *Populus*. Other than *PagGRF1*, several other target genes predicted for PagmiR396d, *PagGRF4*, *PagGRF5*, *PagGRF8*, *PagGRF9*, and *PagGRF10* were also significantly down-regulated with the up-regulation of PagmiR396d expression (Figure 6), which suggests that different *PagGRFs* targeted by PagmiR396d may play redundant and/or independent functions in regulating the secondary xylem formation of “84K” poplar stems. However, in switchgrass (*Panicum virgatum*) with only primary vascular development, the overexpression of *PvMIR396* led to reduced lignin content in stems but the overexpression of *PvGRF9* targeted by PvmiR396 complemented the defective phenotype [47,48]; it was also found that during the transition from primary to secondary vascular development, down-regulated *PtoMIR396* expression and up-regulated *PtoGRF15* expression delayed secondary growth and xylem development in *P. tomentosa* [22]. These findings imply that the down-regulation of miR396 expression may be required for primary vascular development and the up-regulation of miR396 expression may be required for secondary vascular development, and the miR396-*GRFs* module may play an opposite role in regulating the development of primary and secondary xylem (Figure 8).

#### 3.2.2. PagmiR395c-PagGA2ox1/PagLHW/PagSULTR2/PagPolyubiquitin 1 Modules

PagmiR395c-targeted *PagGA2ox1* was significantly up-regulated in the secondary growth of “84K” poplar stems (Figure 6), indicating that PagmiR395c may affect the GA synthesis. GA2ox (GA2-oxidase) is a major GA catabolic enzyme that can deactivate GA in plants [49]. In hybrid poplar (*P. tremula* × *P. alba*), overexpression of *PtaGA2ox1* caused hyperaccumulation of mRNA transcripts and dwarf phenotype [49]; by contrast, silencing of *PttGA2ox* in model plant tobacco (*Nicotiana tabacum* L.) enhanced cambial activity and increased the number and size of xylem fiber cells [50], similar to the phenotype of transgenic poplar that overexpressed the *AtGA20ox* in *P. tremula* × *P. tremuloides* [51]. It is worth noting that in tobacco, overexpression of the *PttGA20ox* resulted in an excessively high activity of the PttGA2ox; moreover, crossing *PttGA20ox* overexpressed plants with *PttGA2ox* silenced plants did not yield additive effects [50]. These findings imply that the *GA2ox* may be feedback-regulated by the GA level and thereby maintain endogenous GA homeostasis in plants. Thus, we suggest that the PagmiR395c-*PagGA2ox1* module plays an important role in regulating the cambial differentiation into xylem and cell expansion by controlling the endogenous GA level in the secondary growth of “84K” poplar stems (Figure 8).

Similarly, we found that the PagmiR395c-*PagLHW* module may also be involved in regulating the cambial differentiation into secondary xylem. As mentioned earlier, LHW was initially found in *Arabidopsis* to participate in the early development of root xylem because the mutation of this gene resulted in reduced xylem cells, thereby giving rise to an abnormal pattern of protoxylem pole, and thus, the LHW is thought to control the number of vascular cells by regulating the size of stem cell pool in primary and lateral roots [24]. In this study, it was found for the first time that *PagLHW* was significantly up-regulated in the secondary growth of “84K” poplar stems, while PagmiR395c, which targeted *PagLHW*, was significantly down-regulated (Figure 6), suggesting that PagmiR395c may be involved in regulating the secondary xylem formation in “84K” poplar stems by targeting *PagLHW* (Figure 8).

MiR395c was found to regulate the formation of secondary cell walls in poplar xylem as a negative regulator [23]. In “84K” poplar, the overexpression of *PagMIR395c* inhibited the biosynthesis of sulfate metabolic products by targeting ATP sulfurylase genes and a sulfate transporter gene *PagSULTR2*;*1*, which led to the reduction of ABA synthesis and then down-regulation of *PagMYB46* expression, thereby resulting in a decrease in the secondary cell wall thickness of xylem fibers and lignin content in stems [23]. In this study, we found that the PagmiR395c was significantly down-regulated, while its target *PagSULTR2* and the ABA synthesis-related DEGs, as well as *PagMYB46,* were significantly up-regulated (Table S3), which is consistent with the previous report. Considering that sulfur nutrition is essential for plant growth and development [52], and SULTRs can transport sulfate to developing tissues or organs to meet the increasing demand [53], we suggest that the PagmiR395c-*PagSULTR2* module regulating the secondary cell wall formation through sulfate metabolism may be attributed to the nutrient transport and allocation required for growing development of secondary xylem (Figure 8).

Xylem vessels and fibers undergo PCD after deposition of the secondary cell walls [54], which is essential for xylem development in plants [55]. The ubiquitin-mediated proteolysis pathway plays an important role in PCD, and in this pathway, ubiquitin is covalently attached to cellular proteins, and then the ubiquitinated proteins are targeted for degradation by the proteasome [56]. In this study, PagmiR395c-targeted *PagPolyubiquitin 1* gene was significantly up-regulated in the secondary growth of “84K” poplar stems (Figure 6), and we thus suggest that the down-regulation of PagmiR395c expression may relieve the inhibitory effect on the expression of the *PagPolyubiquitin 1* gene, thereby promoting the ubiquitin-mediated PCD of secondary xylem cells (Figure 8).

#### 3.2.3. PagmiR482d-PagLAC4 Module

A recent study showed that PtomiR482 may be involved in the regulation of dynamic cambial activity during the active-dormant cycles in *P. tomentosa* by targeting a non-coding RNA [44]. In this study, we found that PagmiR482 targeted a laccase gene *PagLAC4* (a homolog of *AtLAC4*), and the *PagLAC4* was significantly up-regulated with the down-regulation of PagmiR482 expression (Figure 6). Laccases located in the cell walls can polymerize lignin precursors (monolignols) into lignins [57], and several laccases have been confirmed to be involved in the regulation of lignin biosynthesis and secondary cell wall formation in plants [58,59,60,61]. In *Arabidopsis*, the single mutation of *AtLAC4* and mutation combinations with *AtLAC17* and/or *AtLAC11* resulted in reduced lignin content and collapsed xylem cells to varying degrees [58,59]. *PtoLAC14* could functionally complement the phenotype of the *atlac4* mutant, and overexpression of the *PtoLAC14* promoted the lignification of poplar [60]. These findings suggest that LACs have certain functional redundancy in plants, and PagmiR482d is involved in regulating the secondary cell wall deposition in the xylem cells during the secondary growth of “84K” poplar stems by targeting *PagLAC4* (Figure 8).

#### 3.2.4. PagmiR167e-PagbHLH62 and PagmiR167f/g/h-PagbHLH110 Modules

In this study, we found that PagmiR167e was predicted to target *PagbHLH62* whereas PagmiR167f, PagmiR167g, and PagmiR167h were all predicted to target *PagbHLH110*, and when the four PagmiR167 family members were significantly down-regulated, the *PagbHLH62* and *PagbHLH110* showed a significantly opposite expression trend in the secondary growth of “84K” poplar stems (Figure 6). In *Camelina sativa*, overexpression of *CsMIR167a* resulted in the down-regulation of genes associated with lignin biosynthesis in the seed coat development [62], indicating that miR167 is involved in the secondary cell wall formation by regulating the expression of lignin biosynthesis-related genes. Several *bHLH* genes have been confirmed to be involved in the regulation of secondary xylem development in poplar stems; for example, overexpressing *bHLH* genes *PagBEE3L*, *PagUNE12,* or *PtrbHLH186* in poplar resulted in an increase in number of xylem cells and lignin content, as well as the up-regulation of lignin biosynthesis-related genes [63]. Based on the existing evidence, we speculate that PagmiR167e-*PagbHLH62* and PagmiR167f/g/h-*PagbHLH110* modules may contribute to the secondary xylem development of “84K” poplar stems by regulating lignin synthesis and differentiation of cambium into xylem cells (Figure 8).

## 4. Materials and Methods

### 4.1. Plant Materials and Sample Collection

An “84K” poplar was used for all experiments. Stem samples were collected from 1st to 2nd internodes, 3rd to 4th internodes, and 8th to 10th internodes of poplar, respectively. Some samples were used for histochemistry and microscopic observation, and other samples were used for subsequent transcriptome and small RNA analyses. In the experiments, 1st–2nd internodes represent stems that have not yet initiated secondary growth, designated as stage 1; 3rd–4th internodes represent stems that have just initiated secondary growth, designated as stage 2; 8th–10th internodes represent stems that have significant secondary growth, designated as stage 3. For each stage, three biological replicates were prepared.

### 4.2. Histochemistry and Microscopic Observation

Stem samples were cut into small pieces about 0.5 cm in length and fixed in FAA solution containing 5 mL 4% paraformaldehyde, 90 mL 70% ethanol, and 5 mL acetic acid as described by [64]. Then, the stem pieces were dehydrated in a graded ethanol series and embedded in paraffin. Sections of 8 μm thickness were cut with a microtome (Leica RM2245; Leica, Nussloch, Germany). After dewaxing, the sections were stained with 0.1% (*w*/*v*) toluidine blue O (Sigma, St. Louis, MO, USA) for 2 h and then washed with distilled water. Finally, all the sections were observed with a microscope (Leica DM6000B).

### 4.3. RNA Extraction

The total RNAs of stem samples were extracted using the easy-spin Plant RNA Kit (Aidlab Biotech, Beijing, China) following the manufacturer’s instructions. The quality and integrity of the isolated RNAs were evaluated using the Thermo Scientific NanoDrop2000 (Thermo Fisher Scientific, Wilmington, DE, USA) and Agient2100 (LabChip GX, Shanghai, China). High-quality RNAs were used for subsequent sequencing and real-time quantitative PCR.

### 4.4. Transcriptome Sequencing and Analysis

The mRNAs were purified from extracted total RNAs by the interaction of the poly (A) tails and magnetic oligo(dT) beads and prepared to construct cDNA libraries by using a Hieff NGS Ultima Dual-mode mRNA Library Prep Kit for Illumina (NEB, San Diego, CA, USA). After PCR enrichment and quality control, the libraries were sequenced on the Illumina novaseq6000 platform of Biotech (Beijing, China), generating 150-bp paired-end reads. High-quality reads were obtained by removing adapter contaminations and nucleotides with low-quality scores. Subsequently, the cleaned reads were mapped to the reference genome Pop_tri_v3 of *P. trichocarpa* (https://www.ncbi.nlm.nih.gov/assembly/GCF_000002775.4/ (accessed on 22 December 2021)). Fragments per kilobase of transcript per million fragments mapped (FPKM) were used to standardize gene expression levels. Differentially expressed genes were identified by DESeq2 software (v1.6.3) with the criteria of |log2 fold changes (FC)| ≥ 1 and FDR < 0.01. Kyoto Encyclopedia of Genes and Genomes (KEGG) pathway enrichment analysis of identified DEGs was performed using KOBAS.

### 4.5. Small RNA Sequencing and Analysis

Small RNA libraries were prepared from extracted total RNAs using a VAHTSTM Small RNA Library Prep Kit for Illumina (NEB, USA) according to the manufacturer’s protocol and sequenced on an Illumina novaseq6000 platform (Biomarker technologies, Beijing, China), generating 50-bp single-end reads. Low-quality and contaminated sequences (adaptor, reads shorter than 18nt or longer than 30 nt readers, reads without 3′ adapter sequences and the insert) were removed. Then, ribosomal RNAs (rRNAs), transfer RNAs (tRNAs), small nucleolar RNAs (snoRNAs), and the repeated sequences were filtered to obtain unannotated reads containing miRNAs, which were mapped to the reference genome Pop_tri_v3 using Bowtie software (v1.0.0). For the prediction of conserved miRNAs, the mapped reads were compared with mature miRNAs in miRbase (v22) software, and one mismatch in the miRNA sequence was allowed. The remaining unannotated reads were further analyzed by miRDeep2 to predict novel miRNAs. The prediction is based on the miRNA precursors, which have a landmark hairpin-stem-loop structure. Expression of miRNAs in each sample was calculated and normalized by the TPM algorithm. Differentially expressed miRNAs (DEMs) were identified as miRNAs with |log2 fold changes (FC)| ≥ 1 and FDR ≤ 0.01 by using DESeq2 software (v1.6.3).

### 4.6. MiRNA Target Identification and Annotation

The target genes of miRNAs were predicted using the TargetFinder software (v1.6) and were then blasted against NR, Swiss-Prot, GO, COG, KEGG, KOG, and Pfam databases to predict potential functions.

### 4.7. Real-Time Quantitative PCR Analysis

RT-qPCR was used to validate the expression of the DEMs and their targets. Primer pairs were designed using Primer Premier (version 5.0). The RT-qPCR was performed using the SYBR Green PCR MasterMix (Vazyme, Nanjing, China) according to the manufacturer’s instructions. For the DEMs, stem-loop RT-qPCR was performed with U6 as a reference gene. For the DEM targets, RT-qPCR was conducted with UBQ as a reference gene. The final relative expression level was calculated using the 2^−ΔΔCt^ method. The significance was tested by Duncan’s multiple range test at the 5% level. Three replicates were analyzed for each sample and internal reference. All the primers used for RT-qPCR are listed in Table S9.

## 5. Conclusions

We first ascertained three representative stages of pre-initiation, initiation, and post-initiation of secondary growth in “84K” poplar. Thereafter, we provided a comprehensive regulatory network for secondary growth, especially the onset of secondary growth, using joint analysis of transcriptome and miRNAs, and a large of DEGs and DEMs involved in secondary xylem formation were identified. Furthermore, miRNA-target modules with significant opposite expression tendencies were identified, and the underlying mechanisms by which these miRNA-target modules regulate cambial differentiation into secondary xylem and subsequent cell expansion, secondary cell wall deposition, and PCD were revealed. The RT-qPCR validated the reliability of sequencing results. Our findings provide new insights into the mechanism of secondary xylem formation and broaden our understanding of secondary xylem development.

## Data Availability

The raw sequence reads of transcriptome and small RNAs in this study have been deposited in the National Center for Biotechnology Information (NCBI) BioProject database under accession number PRJNA1013908 and PRJNA1014995.

## Supplementary Materials

The supporting information can be downloaded at: https://www.mdpi.com/article/10.3390/ijms242216438/s1.
