# Supplementary material for: Transcriptome and miRNAs Profiles Reveal Regulatory Network and Key Regulators of Secondary Xylem Formation in “84K” Poplar
Source: Int J Mol Sci. 2023 Nov 17;24(22):16438. doi: 10.3390/ijms242216438 (PMC10671414; doi:10.3390/ijms242216438)
Supplement: Supplementary file 1 [file ijms-24-16438-s001.zip › Supplementary Figures S1-S3.pdf]

Supplementary Figure S1

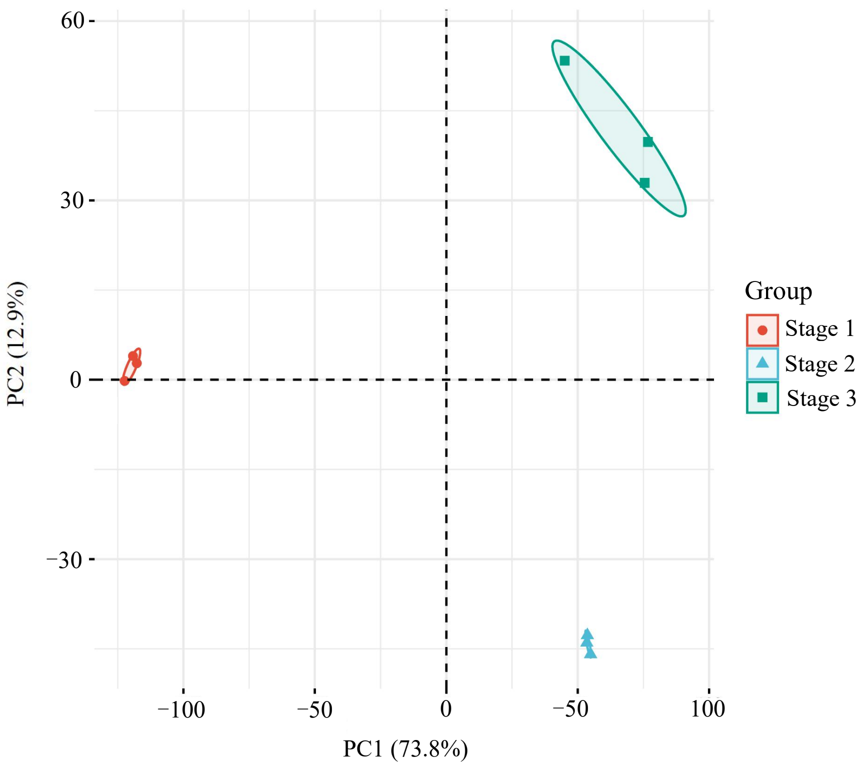

Figure S1. Principal component analysis for transcriptome comparison from three developmental stages of "84K" poplar stems.

Supplementary Figure S2

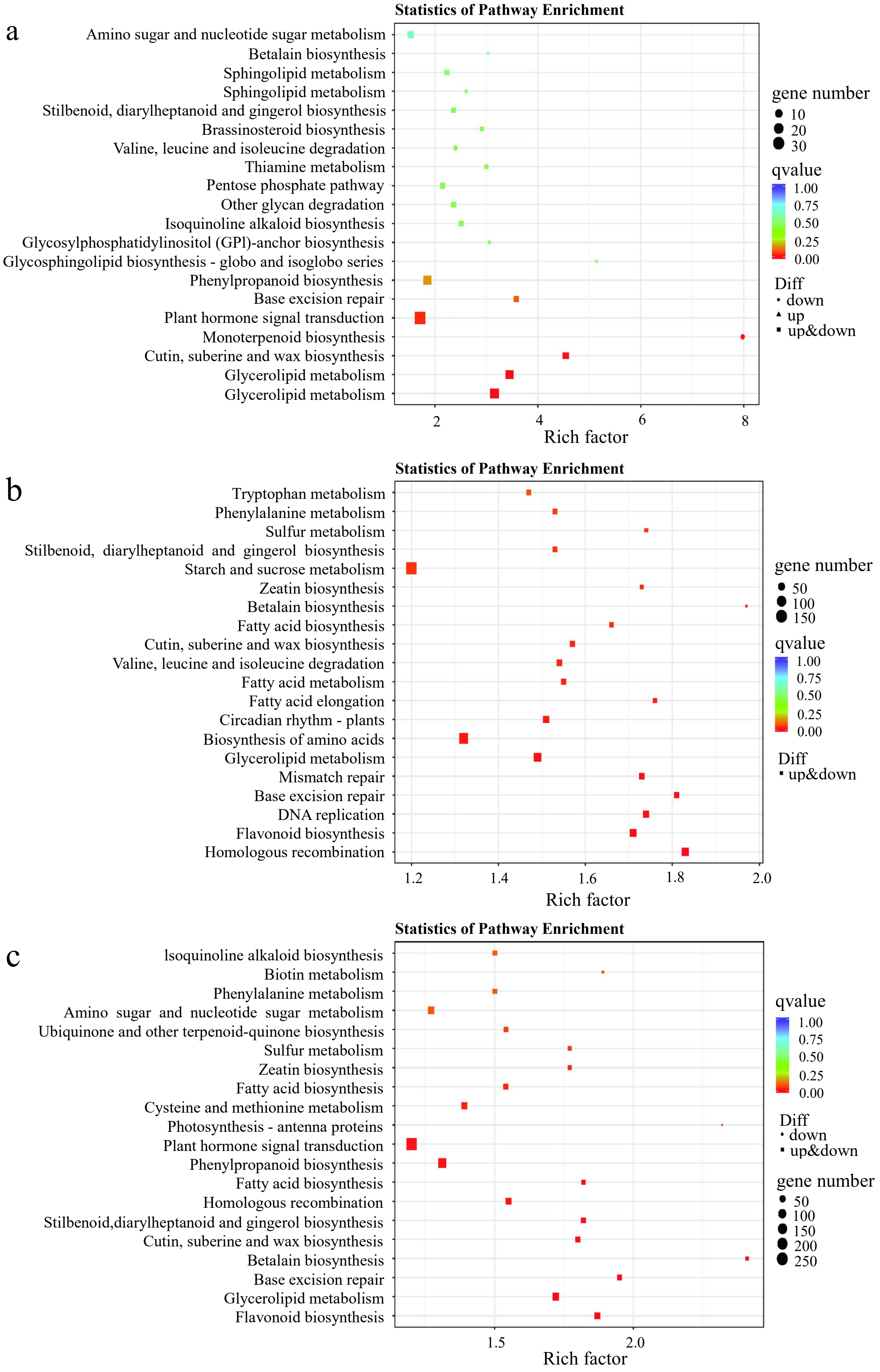

Figure S2. Enrichment of all differentially expressed genes (DEGs). High and low values are showed in red and green, respectively. (a-c) indicate the stage 1 versus stage 2, stage 2 versus stage 3, stage 1 versus stage 3 DEGs pairwise group, respectively.

Supplementary Figure S3

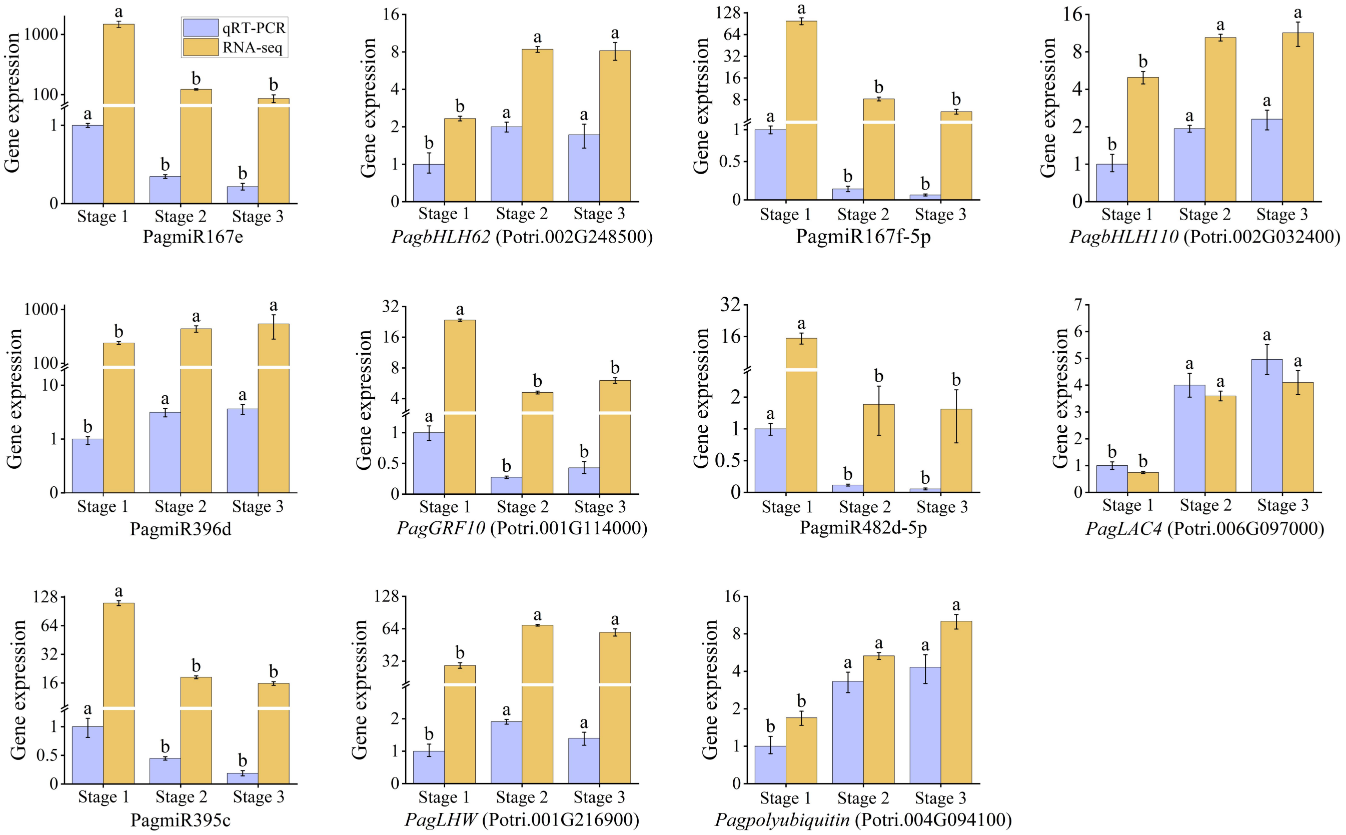

Figure S3. Validation of selected differentially expressed miRNAs (DEMs) and their targets by RT-qPCR. All data in the figure represents the mean values of three independent experiments ± standard deviation (SD) (n = 3). Different letters above the columns indicate significant differences at $P < 0.05$.
