# Supplementary material for: Transcriptome and miRNAs Profiles Reveal Regulatory Network and Key Regulators of Secondary Xylem Formation in “84K” Poplar
Source: Int J Mol Sci. 2023 Nov 17;24(22):16438. doi: 10.3390/ijms242216438 (PMC10671414; doi:10.3390/ijms242216438)
Supplement: Supplementary file 1 [file ijms-24-16438-s001.zip › Supplementary Table S1.pdf]

Supplementary Table S1. Summary of RNA-Seq data and mapping statistics

Libraries	Cleaned reads	Q30(%)	Mapped reads	Ratio	GC content(%)
Stage 1-1	63,415,856	94.06%	52,850,270	83.34%	44.63
Stage 1-2	61,464,330	92.96%	51,165,407	83.24%	44.58
Stage 1-3	59,603,792	94.63%	48,746,229	81.78%	44.58
Stage 2-1	69,058,260	94.44%	57,510,085	83.28%	44.42
Stage 2-2	74,835,696	94.18%	62,274,226	83.21%	44.24
Stage 2-3	61,717,326	94.10%	51,175,131	82.92%	44.41
Stage 3-1	64,791,676	94.17%	53,728,962	82.93%	44.36
Stage 3-2	62,609,344	94.21%	51,813,026	82.76%	44.57
Stage 3-3	63,542,114	94.06%	52,754,093	83.02%	44.38
Total	581,038,394	--	482,017,429	82.96%	--

Q30 (%): bases with a quality value > 30; Ratio: the ratio of mapped reads to cleaned reads
