# Supplementary material for: Transcriptome and miRNAs Profiles Reveal Regulatory Network and Key Regulators of Secondary Xylem Formation in “84K” Poplar
Source: Int J Mol Sci. 2023 Nov 17;24(22):16438. doi: 10.3390/ijms242216438 (PMC10671414; doi:10.3390/ijms242216438)
Supplement: Supplementary file 1 [file ijms-24-16438-s001.zip › Supplementary Table S5.pdf]

Supplementary Table S5. Statistics of small RNA-Seq data and mapping

Libraries	Cleaned reads	Q30(%)	Mapped reads	Ratio
Stage 1-1	4,247,416	96.63	2,647,418	62.33%
Stage 1-2	4,741,252	96.58	2,941,253	62.04%
Stage 1-3	5,302,185	96.57	3,502,185	66.05%
Stage 2-1	6,131,877	96.27	3,682,726	60.06%
Stage 2-2	5,852,742	96.29	3,540,676	60.50%
Stage 2-3	4,656,548	95.31	2,856,549	61.34%
Stage 3-1	5,829,718	95.9	3,829,719	65.69%
Stage 3-2	9,502,370	96.72	6,366,465	67.00%
Stage 3-3	4,968,264	96.22	3,079,293	61.98%
Total	51,232,372	--	32,446,284	63.33%

Q30 (%): bases with a quality value > 30; Ratio: the ratio of mapped reads to cleaned reads
