# Supplementary material for: Transcriptome and miRNAs Profiles Reveal Regulatory Network and Key Regulators of Secondary Xylem Formation in “84K” Poplar
Source: Int J Mol Sci. 2023 Nov 17;24(22):16438. doi: 10.3390/ijms242216438 (PMC10671414; doi:10.3390/ijms242216438)
Supplement: Supplementary file 1 [file ijms-24-16438-s001.zip › Supplementary Table S8.pdf]

Supplementary Table S8. Putative orthologs of transcription factors involved in the secondary xylem formation.

transcription factors	putative "84K "poplar orthologs	Reference
PtrWOX4a (Potri.014G025300)	PagWOX4 (Potri.014G025300)	Hou et al., 2020 [19]
PtrTCP20 (Potri.001G096600)	PagTCP20 (Potri.001G096600)	Hou et al., 2020 [19]
AtNST1 (AT2G46770)	PagNAC043 (Potri.014G104800)	Sun et al., 2022 [2]
AtNST2 (AT3G61910)	PagNAC043 (Potri.002G178700)	Sun et al., 2022 [2]
AtSND1 (AT1G32770)	PagNAC043 (Potri.001G448400)	Sun et al., 2022 [2]
AtLHW (AT2G27230)	PagLHW (Potri.001G216900)	NCBI
AtMYB46 (AT5G12870)	PagMYB20 (Potri.017G130300)	Xiao et al., 2021 [28]
AtMYB83 (AT3G08500)	PagMYB20 (Potri.017G130300)	Xiao et al., 2021 [28]
AtTCP13(AT3G02150)	PagTCP13 (Potri. 017G094800)	NCBI
AtKUA1 (AT5G47390)	PagKUA1 (Potri.010G039300)	NCBI
PtoGRF15 (Potri.014G012800)	PagGRF1 (Potri.014G012800)	Wang et al., 2023 [22]
PtrLAC4 (Potri.006G096900)	PagLAC4 (Potri.006G096900)	Lu et al., 2013 [61]

Q30 (%): bases with a quality value > 30; Ratio: the ratio of mapped reads to cleaned reads
